# Immigration, Domination, and ‘Proportional Patriotism’: Recovering the Sociology of Herbert Adolphus Miller

**DOI:** 10.1007/s12108-022-09530-7

**Published:** 2022-03-19

**Authors:** Jan Balon, John Holmwood

**Affiliations:** Centre for Science, Technology, and Society Studies, Institute of Philosophy CAS, Jilská 1, 110 00 Praha 1, Czech Republic

**Keywords:** Americanization, Immigration, Cleveland school survey, Carnegie corporation

## Abstract

This article addresses the sociological approach and political engagements of the early twentieth century sociologist, Herbert Adolphus Miller (1875–1951). He is now largely forgotten, but he had deep connections within the Chicago milieu of pragmatist sociology and social reform activities through both the Settlement movement and the Survey movement. In 1914 he wrote a volume in the Cleveland Survey on Immigrant children in the school system and in 1918 was appointed to head the division on Immigrant Contributions in the Carnegie Corporation’s project on ‘Methods of Americanization’, in which Robert E. Park was head of the division on Immigrant Press and Theater (Park in *The Immigrant Press*, 1922). If Miller’s name is recognized at all it is as author with Park of *Old World Traits Transplanted* (1921), a work subsequently attributed to W. I. Thomas. We examine the nature of Miller’s research on immigrant populations from subject nationalities in Europe, undertaken in Cleveland and as part of the Carnegie project. He left the latter project mid-way through to become part of a small group that drafted the Czechoslovakian Declaration of Independence in November 1918. We show how Miller developed a distinctive approach to ‘Americanization’ through his idea of ‘proportional patriotism’ that challenged the dominant discourse of assimilation that became entrenched in the years after the end of the first world war and which was largely accepted by Park and by Thomas. He was dismissed in 1932 from Ohio State University because of his views on race mixing and his criticisms of the British and Japanese empires.

“Pragmatism insists that the truth about phenomena inheres in the way things work. If we look at the chaotic world about us we shall see that social philosophy has been largely dominated by theories that are not working. As we make practical adjustments to truth discovered in action, we may hope to substitute progress for chaos” (Miller, 1924, p. xvii).

Herbert Adolphus Miller (1875–1951) is a marginal, and largely forgotten, figure in the history of sociology. He exists on the radar as someone who carried out some important research on immigrants in the US and was a dedicated supporter of Afro-American emancipation. Through his political engagement especially during WWI, he also made important contributions to the political independence and representation of various dominated nationalities and subject groups in Central and Eastern Europe. He does not feature in most histories of sociology, except, at best, as a footnote, and as someone who did not quite make the cut. Indeed, his main claim for recognition has come to be denied. Floyd House, for example, in his *Development of Sociology*, notes in relation to a discussion of the work of W. I. Thomas, “it is an open secret that Old World Traits Transplanted, published over the signatures of Robert E. Park and H. A. Miller, was the product, chiefly, of the work of Thomas” (House, 1936, p. 284)^1^.

Miller’s objectives were rooted in pragmatist philosophy. For example, his PhD at Harvard between 1902 and 1905 was undertaken with Josiah Royce and William James. He was also active within the settlement movement, and progressive politics more generally, alongside others influenced by pragmatism. Jane Addams had established the first settlement at Hull House in 1889 and, together with Graham Taylor in 1897, set up the Chicago Commons, serving immigrant populations (Lissak, 1989; Schneiderhahn, 2011). Miller was involved with the latter and was also closely connected with Mary E. McDowell who established the University of Chicago Settlement House in 1894 (Lissak, 1989). Many of the Chicago settlement women were more radical on issues of race than was typical of the men of Chicago sociology, with Addams, in particular, treating race relations as involving oppression and domination (Diner, 1970), as did Miller. In this respect, we can suggest that Miller was an early contributor to a ‘conflict sociology’ with roots in pragmatism that would become more familiar from the writings of C. W. Mills, albeit a concern with issues of race was absent from the latter’s approach^2^.

Miller had taught at Fisk University prior to his PhD studies at Harvard (Wright, 2010), where he met his future wife Elizabeth Cravath, daughter of the first president of the university, Erastus Milo Cravath. This would be the basis of a lifelong association with Fisk and, indeed, its most famous alumnus W.E.B. Du Bois. This experience was reflected in his choice of PhD topic which was one of the first applications of eugenics (Miller, 1914b) to show that differences among the races was founded in prejudice, rather than biology. For reasons of space, we will present this aspect of Miller’s work in a separate article.

Miller spent a significant part of his career – between 1914 and 1924 – at Oberlin College in Northeast Ohio, itself a racially integrated college under the auspices of the American Missionary Association, albeit that its policy of racial mixing was in serious decline (Smith, 2016)^3^. W. I. Thomas had himself taught at Oberlin for two academic years in 1894 and 1895 before moving to Chicago. Miller appeared to be well-networked – he met Thomas in 1911, shortly before Thomas met Park. According to a note appended to the box of his papers held at Temple University, Miller took a course with Thomas at Chicago in 1911 who also arranged for him to visit Bohemia in 1912 ‘to study an alien culture’^4^. In a letter to Kimball Young about his name being removed from *Old World Traits*, Thomas was unforthcoming about the circumstances, but clear about his relationships with Park and Miller, that they “were my friends and acted friendly”^5^. Yet, Park, Thomas and Miller moved apart.

Thomas had taken over responsibility for the division on Immigrant Contributions within the Carnegie Corporation project on Methods of Americanization from Miller in November 1918, with *Old World Traits* published in 1921. In 1924, Miller moved to Ohio State University in Columbus, which was, at that time, the largest sociology department in Ohio. This coincided with Miller’s publication of his book, *Races, Nations and Classes* (Miller, 1924) which drew upon papers he had written during his time as director of the division on Immigrant Contributions. As we will see, Miller developed a distinctly more radical position than that of his erstwhile colleagues Park or Thomas, introducing the ideas of domination and oppression into the discussion of relations among races and ethnic minorities.

There is some suggestion of disquiet about Miller’s political involvements and pedagogic practices at Oberlin (or, at least, knowledge of them followed him to Ohio State)^6^. In 1931 moves began to dismiss him from Ohio State in a case that was taken up by the American Association of University Professors (Sabine, 1931). He was dismissed in 1932. He undertook visiting lectures for a fee organised on his behalf by the Speakers’ Forum of Columbus – an adjunct of the Foreign Policy Association, of which Miller was an early and active member – for a year before his appointment at Bryn Mawr in 1933, where he taught sociology in the department of social economy until his retirement in 1940. Thereafter, he held a number of visiting positions, including a year at Temple University and at Beloit College and Pennsylvania State College (which became Pennsylvania State University in 1953), hired on a semester-by-semester basis. He ended his career at Black Mountain College in Asheville, North Carolina, where he taught between 1943 and 1947. Black Mountain was set up on ‘Deweyan’ principles of education as an experimental artists community and college in 1933 after the dismissal of John Rice from Rollins College (Reynolds, 1998).

In short, Miller was connected with noteworthy educational institutions in the United States – from the Ivy League through historically Black colleges and progressive liberal experiments. Through it all, he was active in professional sociological organisations. These included the Ohio Sociological Society (of which he was president in 1932, the year of his dismissal from Ohio State University, though that event is not mentioned in its journal)^7^ and the American Sociological Society and was chair of several of its committees (including one on international relations) and member of the executive committee at the time of his sacking. Throughout his career he was active in the meetings of both societies, discussing substantive matters relating to race and immigration as well as sociological pedagogy. He was also founding member of other associations involved with international relations, most specifically the successor organization to the League of Free Nations Association, the Foreign Policy Association, (where he was a critic of empire, including British empire, one of the reasons given for his dismissal from Ohio State University). Yet, his contribution is lost to the history of sociological thought and its connections with pragmatism’s promise of public education for democracy.

In this article, we revisit Miller’s activities in two important projects that were carried out during and shortly after World War I, namely the Cleveland survey and the Carnegie Corporation’s Americanization Studies. Both projects are now largely excluded from the histories of sociological research. Yet we think that Miller, in the process of working on the concept, or method, of ‘Americanization’, made a remarkable and significant contribution to the study of immigration and understanding of races, nations, and classes.

## The Survey Movement in the Development of Sociology

Miller moved to Oberlin in 1914 where he became involved in the Cleveland survey. This was initially planned to be similar to the Pittsburgh survey which had been begun in 1907 (Cohen, 1991). The latter was directed by the ‘social entrepreneur’, Paul Kellogg who had also become editor of the magazine, *Charities and the Commons*, in which the surveys were published in a series of 35 articles between January and March 1909, and subsequently in other books and articles up to 1914 (Chambers, 1971; Cohen, 1991)^8^. The publications in *Charities and the Commons* became the occasion for a change of its name to *The Survey*. It would become an important venue for the publication of Miller’s work under the auspices of the Carnegie project and for social research and the promotion of social reform more generally.

The Pittsburgh survey is widely seen as an important moment in the development of social research. However, Bulmer (1996), for example, also argues that it marked the moment when sociology in the academy turned away from research embedded in reformist aspirations to become more rooted in ‘professionalism’ and that it was, “orthogonal to academic sociology, rather than a linear ancestor” (Bulmer, 1996, p. 15). On this understanding, Miller hitched his career to a wagon that was on a different path even before he became part of the very Carnegie research project that provided a different trajectory for Park and Thomas. Yet, as Furner (2000, p. 407) suggests, it was politics (and gender and race), not *professionalism*, that separated out ‘survey’ reformers. And, of course, by that token, academic sociology was not without its own ‘politics’ even if it was suppressed. Nor could it exclusively claim the mantle of scientific professionalism, as we shall show in the example of Miller. We shall also show that that the Survey movement remained important to the early development of Chicago sociology precisely because of the way in which the Carnegie project was an integral part of the movement.

In January 1914, Fred Goff set up the Cleveland Foundation as a public trust institution with an obvious aspiration to avoid the vulnerabilities of big private philanthropic organisations and their corporate trustee structure. The risks of ‘capture’ were serious. Only a year after the official establishment of the foundation, Goff had to go to New York to defend its position at the hearings of the Federal Industrial Relations Committee. In the aftermath of the Ludlow Massacre on the Colorado coalfields^9^, all charitable foundations had come under suspicion on the grounds that they might represent an effort to “perpetuate the present position of predatory wealth through the corruption of the sources of public information^10^.” The coal-mining company involved in the Ludlow Massacre was owned by the Rockefeller family and this cast suspicion on the Rockefeller Foundation and its interest in funding studies on labor unrest. Goff’s community-based institutional model provided an opportunity to fend off such allegations and held up a different understanding of research, civic debate and reform, one aligned with the aspirations of pragmatism and distinct from the orientation to business efficiency that Mead (1899) had also identified as a new problem to be confronted.

At the outset, Goff envisioned an all-encompassing municipal survey similar to that for Pittsburgh. The Cleveland Foundation hired Allen T. Burns as the paid director of a five-member Survey Committee. Burns was himself a significant figure. He was licensed as a Baptist minister, but turned toward social action and involvement in the settlement movement at Chicago Commons from where he was appointed first Dean of the Chicago School of Civics and Philanthropy between 1907–1909 (Friends & Relatives of Allen T. Burns, 1954, pp. 5–6). The School had been established under a different guise in 1903–1904 to teach the first courses in social work associated with the University of Chicago. The relationship was fraught and the university withdrew support in 1905 when it continued under the auspices of the Chicago Commons. It was finally incorporated into the university in 1920, with the Chicago sociologists maintaining their distance. Burns left the University of Chicago in 1909 to become secretary of the Pittsburgh Civic Commission with administrative responsibility for the Pittsburgh survey conducted by Paul Kellogg (Cohen, 1991). He then moved to the Cleveland Foundation in 1914 as Director of Surveys with the intention of setting up surveys similar to those of Pittsburgh (Tittle, 1992). However, the Committee eventually put a halt to Goff’s original extensive plan and opted for “investigating one topic of societal interest at a time” (Tittle, 1992, p. 48). At the same time, the orientation to ‘business efficiency’ came to dominate over Goff’s initial aspirations (Miggins, 2014).

## Schooling the Immigrant

The Committee identified three major topics: public education, recreation and the administration of justice. A number of surveys were devoted to each topic. High drop-out rates from schools called for immediate action in educational matters. Goff and Burns hired the director of the Russell Sage Foundation’s division of education, Leonard Ayres, a statistician who had made a name for himself as a proficient organizer of large-scale studies. The Cleveland school surveys were, by far, the largest ever done of a public school system at that time. Twenty-four surveys and a summary were published, with Miller appointed to conduct one on the problems of immigrant children within the school system. Ayres’s approach epitomized a notion of reform through research in a bid to pin down principal factors that “determine the quality of results and efficiency of work of a school system” (Ayers, 1917, p. 49). In consequence, his notion of school reform rested on measurable factors and turned the spotlight on the criterion of ‘efficiency’ in educational matters.

The Survey Committee, dominated by businessmen, initiated a number of public debates on the recommendations for action. The debates voiced recurrent criticism that the business department was forcing out the educational work and excessively dominated the school system. In a response, Ayers (1917, p. 57) maintained that “the fact remains that the business management is so markedly efficient that it constitutes one of the real and important assets of the educational situation.” Ultimately, ‘the search for the one best system’ (Miggins, 1986) concluded that the way to overcome the educational troubles would be through the reinforcement of business models that would ‘promote efficiency’ and ‘facilitate uniformity’. This tension between ‘social survey activism’ and established local interests, and the victory of the latter is something also described for Pittsburgh by Turner (1996). The victory of the same interests in Cleveland would be exemplified by Ayres’s large-scale re-organisation of the public school system in the name of efficiency on the basis of the surveys.

Ayres’s summary report made no reference to Miller’s contribution, *The School and the Immigrant* (Miller, 1916). Miller saw educational problems as reflecting wider social pathologies related to the issues of immigration. In the Cleveland area, the rapid demographic change and large concentrations of urban immigrants had palpable effects on racial, gender and social class divisions. On Miller’s understanding, efficiency measures represented a problematic response as they stood in the way of reforms based on an understanding of the needs of marginalized communities. The issue for Miller was not how to secure integration and uniformity, but how to use the cultural heritage of immigrant groups to facilitate their participation and achievement within the school system. His contribution demonstrated high drop-out rates from ‘steamer classes’ (designed to teach English to recently-arrived children and adults) as well as underachievement in schools. He found the failure of the adult English classes puzzling because such classes had proven successful in recently incorporated American territories – for example, in the Philippines and Porto Rico (*sic*) (Miller, 1916, p. 76). The problem was a failure of action and an absence of a proper curriculum and training for the teachers (Miller, 1916, pp. 83–84)^11^. But, if Cleveland had the highest proportion of non-English speakers compared with other large cities (31% against 23% in New York, for example – Miller, 1916, p. 22), this was not an argument directed only at language. It was also an argument to facilitate naturalization and participation in political life.

Miller’s dictum (in both research and in teaching) taken from pragmatism was the importance of talking to others and understanding their perspective. The dominant approach to Americanization had not allowed for different cultural identities and only reinforced ‘segregation’ by pathologizing what were, for Miller, ‘normal tendencies’. He maintained that “to blame immigrants for their own segregation is unjust” (Miller, 1916, p. 56). Families did not wish their children to ‘grow away’ from them, and it was precisely when they did that a problem of ‘disorganization’ arose. Thus, Miller wrote, “as a matter of fact, we find within these various colonies a neighbourliness and social organization sadly lacking in much of our modern society. A teacher should know something of the social life to be found within these various groups, both in order that she may understand her pupils better and that she may be able to use these social forces to the advantage of the school and the community” (Miller, 1916, p. 56). Instead of invoking patriotic sentiments for public schools, he argued that “in order to understand the social and educational problems of the different foreign groups, it is necessary to study their origin and history” (Miller, 1916, p. 54). Many of the immigrants, Miller argued, “come from subject races and they come here primarily for freedom” (Miller, 1916, pp. 55–56).

In addition, he recommended teaching of ‘home’ languages and the use of curriculum material from ‘home’ cultures, something that would be developed within the Cleveland Library system. This would mitigate the poor achievements of foreign-language-speaking children in schools and their high drop-out rate. It was also important because many of the immigrant groups came from countries where attempts had been made to impose another language upon them (Miller, 1916, p. 42). Paradoxically, for Miller, the teaching of home languages and cultures would facilitate Americanization, once the latter was properly understood and associated with the ‘spirit of freedom’ that had motivated migration. He also recommended that public schools could be used out of hours by the different national groups to encourage both self-expression and identification with the school system.

The aspiration to change the Cleveland educational system – according to Miller, *it was it that was disorganized*, not the immigrant communities – had a practical element that aligned him with library reformers and public-school educators who had an understanding of the special needs of the ‘new’ immigrants. These were often women steeped in the social reform traditions of the settlement movement. According to Jones, “their mission was not to instill American ideas and ideals into the immigrants but to teach immigrants to read and speak the English language and to prepare them for the required citizenship exam, an approach that was pragmatic rather than political” (Jones, 2013, p. 251). A significant figure in library activism, Eleanor Ledbetter, who was a member of the Cleveland Americanization Committee, and later took part in the Carnegie research on Americanization, referred to Miller as “probably the best informed man in the country regarding immigrant backgrounds” (Jones, 2013, p. 259). She looked to him as a mentor for a different approach to Americanization.

The tensions between business efficiency and progressive reform came to a head in Cleveland when Burns was forced to resign in 1917 after political opposition to another report that had seemed to uncover the existence of a new red-light district and which became part of a mayoral electoral battle (Tittle, 1992, p. 78). Burns undertook an evaluation of some settlement houses that were supported by the Carnegie Corporation before he was asked to administer a new Americanization studies programme under its auspices, to which he would appoint Miller in 1918. Clearly, Burns valued his approach and contribution to the Cleveland school survey, yet Miller’s contribution otherwise has been side-lined. There are only sporadic references to it in the histories of educational reforms in America. His colleague in the Carnegie Americanization project, Frank Thompson, made only one brief mention in his report on *Schooling of the Immigrant*, referring to it simply as being ‘mainly critical’ (Thompson, 1920, p. 54). Thompson grounded his own study in the ‘official’ attitude toward education: “it is to our credit that in our schools we have never made invidious comparisons with respect to the children of the immigrant; we have received them on a basis of equality and made them feel that there were no distinctions on account of accidents of birth and economic condition” (Thompson, 1920, p. 73). Miller had provided extensive evidence to the contrary in Cleveland. He was responsible for research on ‘immigrant contributions’ and yet there would seem to be no interaction between him and Thompson^12^. This indicates, perhaps, that the divisions that had beset the Cleveland studies would also become evident within the Carnegie project.

## The Carnegie Americanization Project

The Carnegie Corporation research project on Americanization was initially mooted with Burns in November 1917 and approved by the Board to begin the process of recruitment of research staff in January 1918. Burns divided it up into ten themes, or Divisions, each with a Division Head and all appointed by March 1918. The intention was for it to run for eighteen months with reports delivered by December 1919. Miller was given responsibility for a report on Immigrant Contributions (later to be re-named Immigrant Heritages) with Robert Park responsible for the Division on the Immigrant Press and Theatre (his report was published in 1922)^13^. A final summary report by Burns as Director of the Project, was planned but not published, though he published several talks, including one on ‘American Americanization’ (Burns, 1920). Here he argued for policies different from those associated with the forced ‘Germanification’ of minority populations in those parts of Europe subject to German domination, which he likened to some approaches to Americanization. He also promoted examples of self-organisation of immigrant communities in America^14^. The invitation for the talk from the Education Department of the Municipal Court in New York cites his role as Director of the Carnegie Corporation’s Americanization project, but Burns, himself, makes no mention of any of its studies.

The problem, as far as it can be discerned from the files of the Carnegie Corporation, was that there were delays involving four of the reports with one of them – William M. Leiserson’s report on Industry and the Immigrant – not cleared for publication by a small group of reviewers until May 1923^15^. Most of the reports had been delivered by their due date of December 1919, with the remainder delivered by April 1921. But the review process and publisher delays held up the completion of the series. The contract with the publisher, Harper and Brothers, had been written on terms problematic to the Corporation and placed too much reliance on a marketing strategy that favoured a staged release of titles to maintain public interest, at the same time as public opinion was shifting. Burns was not paid by the Corporation after April 1921 and yet was responsible for supervising the remaining volumes to publication, alongside his responsibilities in his new field of work. He was unable to write his summary until the series would be complete and was anxious to do so^16^. He was working on it up until January 1924 when he was told that the series would be closed without his summary volume, despite it having been advertised in all the series notifications.

In hindsight, it was not just Miller whose reputation faded; that seems also to be the fate of the Carnegie project itself, and Burns too^17^. Their Americanization studies are not widely remarked in the secondary literature on US immigration policy, which is much more concerned with the xenophobic Dillingham Commission and its 42 volume report published in 1911 (King, 2000; Mirel, 2010). The major study of Americanization as a public policy by Hartmann (1948), has just two very brief mentions of it, notwithstanding that the author identifies two moments of intense political concern; specifically, the last decades of the nineteenth century leading up to the Dillingham Commission and 1915–16, when war in Europe had accelerated the pressures to inculcate patriotism^18^. The Dillingham Commission had recommended stringent measures for controlling against poor ‘stock’, promoting eugenic arguments about ‘unfit races’, and arguing for a rigorous assimilation to ‘American values’, actions which were already in train in many cities and were gathered under the proselytizing efforts of the North American Civic League for Immigrants. Different cities had ‘Americanization Committees’ (Hartmann, 1948) and incorporated educators, librarians and social workers under that drive, as we have seen, but not always as willing participants (Jones, 2013).

An emphasis on assimilation is evident in the title of the Carnegie project - ‘The Study of Methods of Americanization or Fusion of Native and Foreign-Born’ – albeit that the focus on identifying *appropriate methods* of Americanization potentially steered away from identifying *social problems* associated with immigration (Lagemann, 1989). At the same time, it gave scope to researchers with a different orientation, like Miller, implicitly to contest the very idea of Americanization through a critique of the current methods used by the many city committees on Americanization that had been formed, just as he had done in his discussion of schooling in Cleveland. Yet, the Carnegie project appears to have missed its moment. By the end of 1918, the impetus for it was petering out. It had been conceived during a period of febrile public concern about the patriotism of immigrants to the USA in the light of possible American entry as a combatant in WW1. Entry in early 1917 was decisive in bringing the war to an end in November 1918 with allied victory well before the publication of any of the studies, which were delivered into a very different political environment. After the war, public opinion settled into support for what Burns (1920) had called ‘German Americanization’, rather than the ‘American Americanization’ he advocated and it had hardened against a more progressive policy, in part as a consequence of the ‘race riots’ that occurred in 1919 in a number of cities, including Chicago (Waskow, 1966).

It has to be admitted that the Carnegie Project no longer holds much interest, except perhaps for historians of the Chicago school, and, even then, it is superseded in importance by the publication between 1918–20 of the 5 volume *Polish Peasant in Europe and America*, by W. I. Thomas and Florian Znaniecki (1996[1918–1921]), which has come to be regarded as a landmark in US sociology (Abbott & Egloff, 2008). *Old World Traits*, it would seem, is merely a transposition of ideas and methods from the *Polish Peasant* to a broader set of immigrant groups, albeit on a significantly reduced canvas. The identification of Thomas as the major contributing author of *Old World Traits* served to reinforce his standing at the same time as attention was directed toward the *Polish Peasant* study as his principal achievement*.*

The Carnegie Corporation itself also shifted its direction of effort away from a direct engagement with social and public policy toward funding the professionalization of social research, following the appointment of a new Director, Frederick Keppel in 1923 (Lagemann, 1989). Significantly, this involved funding a new Social Science Research Council which had been set up with the involvement of Chicago social scientists in 1923. It was also funded by, and consolidated the activities of, other foundations, such as the Rockefeller foundations, and Russell Sage. It became active in promoting appropriate methodologies for social science involving critical appraisals and symposia on exemplary studies, including, in 1939, a volume on *The Polish Peasant* by then Chair of the Chicago Department of Sociology, Blumer (1939). This rehabilitation of W. I. Thomas and his ‘re-incorporation’ into the post-1920 Chicago School continued with an SSRC-funded Committee on W.I. Thomas’s Contributions to Social Science which published a volume of his papers on social behavior and personality (Volkart, 1951) with collateral damage to Miller’s reputation.

The Volkart volume published the concluding chapter from *Old World Traits,* where it was announced that the book, “while published over the names of Park and Miller, was primarily the work of Thomas” (Volkart, 1951, p. 259), confirming the claim of House cited earlier. The date of publication has some significance, albeit unremarked – it was the year Miller died. Volkart referred readers to a confirmatory letter from Allen T. Burns^19^. It is widely understood that the reason was the scandal associated with Thomas’s name. Gordon (1975) in his review of the re-publication of the series of Carnegie studies devotes much of his review to *Old World Traits.* The repressed attribution of authorship is finally rectified on the title page, but the book itself, he suggests, is only of historical interest. He writes that it was before its time in its opposition to ‘racist ideas’, but by the 1970s it was fully assimilated, and “merges imperceptibly with the climate of thought and opinion which produced the civil rights movement of the 1950s, ‘60 s and ‘70 s” (Gordon, 1975, p. 473).

Gordon’s judgement is rather sanguine in the context of what Thomas had written in the final chapter of *Old World* Traits, which is reproduced in the Volkart volume. This included a description of the ‘material value’ found in the use of forced labour and temporary migrant labour in America and Germany respectively, where Thomas states that we know that “this attitude has a bad effect both on the aliens and the culture of the group which receives and uses them as mere things. If visitors are disorderly, unsanitary, or ignorant, the group which incorporates them, even temporarily, will not escape the bad effects of this” (Thomas, in Volkart, 1951, p. 263). Thomas goes on, “every country has a certain amount of culturally undeveloped material. We have it, for instance, in the Negroes and Indians, the Southern mountaineers, the Mexicans and Spanish-Americans, and the slums. There is a limit, however, to the amount of material of this kind that a country can incorporate without losing the character of its culture” (Thomas in Volkart, 1951, p. 263). While Thomas opposed ‘quick and complete Americanization’, this is far from the commitment to cultural pluralism evinced by Miller.

## Re-telling the Story

Gordon celebrated the restoration of Thomas as author and described the story as ‘fully told’ in the new introduction to the reprinted volume, which referred to the scandal that had impacted on Thomas’s reputation. However, the scandal had taken place in April 1918 and, seemingly, had not impeded Thomas’s involvement in the project until the point of publication some three years later. There is no record of why the decision to remove his name was taken, but those involved in the studies were in place throughout^20^. The story, however, is far from fully told. Most references to it rely on the memorial to Robert Park written in later life by Winifred Raushenbush in 1979 (she was mother to Richard Rorty, See Gross, 2008). Her account is based primarily on memory, the published writings of Park, and his papers held at University of Chicago and Fisk University, although no archival sources are indicated for her chapter on the Carnegie research project. The episode is not pursued in Fred H. Matthews’s (1977) study of Park.

Winifred Raushenbush was employed by Park in October 1918 as assistant on his Immigrant Press research (and later as a research assistant preparing the data analysis for the report on the Chicago race riot in the six-months prior to its publication in 1921). Her employment occurs at around the time that Miller was leaving and it offers her recollection of its circumstances^21^. She notes (correctly) that Miller withdrew from the project in late 1918, citing a notice in the November *American Journal of Sociology* to that effect, and that he had taken up work for the ‘League of Central European Nations’ (what was more generally known as the Democratic Mid-European Union). He had become director to it and would be based in Washington and Pittsburgh. The latter published its aims at the end of October 1918, though there was considerable activity leading up to that point. What is at issue is how that earlier activity related to the Carnegie study where Miller was Director of the Division on Immigrant Contributions.

Raushenbush describes Park as taking over from Miller, and that, “he knew there would be difficulties. Miller had employed a large staff, started many projects, and spent a considerable share of his $14,700 budget… [Park] probably would not have taken on the study of immigrant heritages if it had not been possible for him to employ his friend William I. Thomas, then living in New York to assist him” (Raushenbush, 1979, p. 88). Although Thomas’s appointment seemingly happened in close proximity to the scandal, there is significant evidence that it was not directly the occasion of it. The newspaper story of Thomas’s arrest was on April 12^th^ and he resigned from the university on April 16^th^. Abbott and Egloff (2008, p. 221) cite correspondence between Thomas and Mrs Ethel Sturges Dummer, who had funded the *Polish Peasant* study, dated April 9^th^, a few days before the incident, describing his appointment to the Carnegie project^22^. Although Abbott and Egloff do not speculate on the reasons for Thomas’s appointment, or to what exactly he had been appointed, they do not seem to be associated with Miller’s role. In fact, the records of the Carnegie Corporation show that Thomas was involved right at the start of the overall project in March 1918 when he was appointed as ‘General Consultant’. The records provide monthly details of salaries and other expenses and his does not seem to have been a salaried position. The project also had an advisory committee that met regularly, but Thomas was not at those meetings (and not marked as absent)^23^. No-one appears to have been perturbed by the scandal associated with Thomas until three years later when his name was removed from the contract of publication.

Although Miller is linked by authorship to Park, his connection otherwise was most clearly with Thomas, as we have suggested. Raushenbush claims that Park took over from Miller as Director of the Division and that he employed Thomas, a claim that is widely accepted. The archive suggests something different. The nature of Thomas’s active role, and its starting date, are clear from the monthly salary record. This sets out the salaries of directors and field officers on each of the Divisions. Miller is listed from March through to November 1918, when both his and Thomas’s names are listed. Thereafter, it is Thomas’s name that appears. Park is listed separately for the Division on the ‘Immigrant Press and Theatre’ (along with Raushenbusch’s name – as it was then spelled – as field officer). Clearly, they were two separate projects with two Directors acting independently of each other. As we have indicated, the name of the Division for which Miller and Thomas were responsible was not initially called ‘Immigrant Heritages’, but ‘Immigrant Contributions’. It only changed when Thomas took over as its Director. The significance of this will become apparent.

Raushenbush comments on the large staff employed by Miller, with the implication that this was a concern for Park. The number of field officers was, however, fewer than on other projects^24^. There is evidence that there were difficulties with some of the field officers that reflect less well on the change of direction. For example, Eleanor Ledbetter, the librarian at Cleveland Public Library who had developed a strong working relationship with Miller through their common interest in the Slavic community in Cleveland, as we have seen, joined the Carnegie project in November 1918 according to the salary records. However, she left in January, citing, according to Jones, “a conversation she had with Helen Horvath, a local Americanization teacher and Hungarian immigrant, about what was involved. Horvath had reacted negatively to what she viewed as patronizing and offensive survey questions proposed by Robert Ezra Park, an associate of Herbert Adolphus Miller on the Carnegie Corporation Americanization study” (Jones, 2013, p. 257)^25^.

A document called ‘Life Histories and Questionnaire’ was produced for the ‘Division of Immigrant Heritages’ (as it was now named) in January 1919^26^. This document was certainly written by Thomas and was likely the document under criticism by Horvath (and Ledbetter). It is made up of three parts. The first sets out a prospectus for the study of behavior, rather than immigrant organizations, and it does so by setting out the kinds of documents relevant to determine values and attitudes associated with ‘four wishes’. The latter are classed as: the ‘desire for new experience’; the ‘desire for security’; the ‘desire for recognition’ and the ‘desire for response’. It also sets out the familiar argument of Thomas concerning ‘disorganization’ – in which he places ‘revolutionary attitudes’ as a form of organization transitional between the old (peasant) culture and the new (American) culture. These arguments appear in *Old World Traits*, representing a direct transfer of arguments (and indeed ethnographic material) from the *Polish Peasant*. The document also sets out material that could be used to represent values and attitudes – letters, diaries, etc., life records prepared by individuals, etc. and existing autobiographies, much as are also presented in the former study. Finally, a proposed life history questionnaire survey is presented, although there is no evidence that it was used and no findings from it are reported in *Old World Traits*^27^.

The alignment with the *Polish Peasant* following Thomas taking over as Director of the Division is comprehensive. What has gone unremarked in discussions of the episode, however, is that this was also associated with Florian Znaniecki who, according to the monthly salary returns, worked as a field officer on the project for six months from January through to July 1919. He replaced Ledbetter whose work for the project – a manuscript on the Jugoslavs (Ledbetter, 1918) – receives just one citation in the final report in a footnote. Significantly, the same is true also for Miller – a manuscript on the Bohemians receives just one citation. While this may give some credence to Raushenbush’s intimation that the direction of the project under Miller was problematic, it is surprising that there is little discussion of the war and nationalism, either in the questionnaire document, or in *Old World Traits* itself. The issue, as we shall see, is not simply the failure to include unpublished drafts, but also material written and published by Miller in the course of the project and with the approval of Allen T. Burns. Indeed, the new questionnaire document has the flavor of a re-orientation of the project in the light of the end of the war.

Evidence for this interpretation is provided by the fact that the advisory committee had called an emergency day-long meeting in February 1919 to discuss a Government Memorandum on Americanization, which had been circulated to the Directors of the Divisions^28^. This began with the comment that “the recent war was the most effective agency for Americanization ever applied in the United States”. It also set out the importance of, “the deep community of interest between the natives and the great majority of the recent immigrants of the United States. Our country’s war for world liberty included the long sought liberty for the peoples from which the most immigrants have lately come.” The Memorandum went on to describe this as epitomized by banners in a Czecho Slovak parade in Cleveland. It also stated that the American government would continue to be involved in the countries from which immigrants came and that this was a source of potential risk in terms of discontent among the immigrant population. It proposed a bureau dedicated to bringing about the ‘co-operation of the foreign born’. The Memorandum, then, was understood by Burns and the advisory committee as a pivot towards methods of Americanization suitable to the peace. This reinforced the position of the North American Civic League for Immigrants and its role within the Bureau of Education, displacing the more moderate orientation of Burns and leading to the Carnegie Corporation’s increasing sense that the project had been misplaced.

## ‘The Spirit of Freedom’

When Ledbetter commented on her concern about the new direction of the now re-described Immigrant Heritages division, she was reflecting an understanding she shared with Miller and derived from their mutual interests and criticisms of the dominant ideas of Americanization. When Miller withdrew from directing the Division he had been involved in Slavic nationalist movements in Chicago and Cleveland for about a decade. This was a consequence of his involvement with the Chicago settlement movement and his friendship with Mary E. McDowell to whom he was most probably introduced by Thomas. The Czech sociologist, Alice Masaryk, had stayed in the University settlement between 1904 and 1905, and her father Thomas (also a sociologist, philosopher and future president of a newly independent Czechoslovakia) was a visitor to the university and to the settlement in 1902^29^, and briefly in 1907, during his lecture tour for Czechoslovak immigrants; Miller first met with them both on his trip to Czechoslovakia in 1912^30^. Together with McDowell, Miller was active in petitioning the Austrian authorities for the release of Alice Masaryk after her arrest on charges of sedition in 1915. Thomas Masaryk was elected President/Chairman of the Mid-European Union when Miller was appointed director and, in his memoir, describes it as “meeting pretty often to discuss all the ethnographical and political problems of the smaller mid-European peoples” (Masaryk, 1927, p. 237, see also May, 1957).

In 1926, at the request of a publisher in Prague, Miller wrote a memoir on his work with the Mid European Union for publication for the local audience. Miller delivered his manuscript in July, 1926^31^, and wrote a letter to Thomas Masaryk asking him whether he would write an introduction. Although there is evidence that the Czech translator continued her work on the manuscript as late as 1928, Masaryk never sent his introduction and Miller’s memoir was never published in the Czech language^32^. The English text contains descriptions of the activities of the Union, as well as extracts from the minutes, alongside his commentary and account of its relation to the Americanization project^33^. Miller describes arriving at Oberlin on the outbreak of the first world war in August 1914 and being told by a Czech friend that many Czechs in the Austrian army would contrive to be captured and enlist in the Serbian army to fight against Austria. He also describes being asked to write his first article (for the *North American Review*) on ‘Nationalism in Bohemia and Poland’ (Miller, 1914a) – “this was my first contribution toward an understanding in America of the deep and unquenchable aspirations of the oppressed nationalities involved in the war” (Miller, 1926, Chap II, p. 2). It also led to him joining committees being established among Czechs in America (and Cleveland in particular) to pursue the national cause. He also met with Mary McDowell to join her campaign to free Alice Masaryk (resulting in her release, although, he stated, “the full credit belongs to Miss McDowell” (Miller, 1926, Chap II, p. 4)^34^.

It is easy to misunderstand Miller’s orientation to the war and to ‘Americanization’ in that context, not least because he seems to have combined a deep distrust of patriotism and a commitment to cultural pluralism with advocacy of the participation in the war of units in the US military drawn from immigrants to the US from oppressed European minorities. Indeed, in his manuscript he describes that, “in my deep abhorrence of war I had not approved of declaring war against Germany” (Miller, 1926, Chap II, p. 4). However, he felt the call that had been made to academics to use their special knowledge to help government in the war effort, but “there seemed such an impractical value to my knowledge that I remember remarking to a friend that so far as I could see, the only contribution I could make in the present crisis was to do as good teaching as I could in the hope that some day it might have some value” (Miller, 1926, Chap II, p. 5).

By autumn 1917, before his appointment to the Carnegie project, in line with his pragmatist pedagogy, he felt that “as a sociologist, I should try to see an army camp which was a distinct social phenomenon” (Miller, 1926, Chap II, p. 5). He contacted the Y.M.C.A to arrange a talk at Camp Sherman in Ohio where 30,000 troops were in training: “Not assuming that I had anything to say to white troops, I wrote asking if they would not like to have me talk to the Negroes since for many years I had taught at Fisk University. I proposed as the title of my talk, ‘How the Germans Treat Subject Peoples’. What I really wanted to acquire was a perspective to show that the treatment of Negroes in this country was not at all unique and thus arouse, I hoped, their enthusiasm” (Miller, 1926, Chap II, p. 5). Instead, he was detailed to speak to white troops, but also to commissioned officers in charge of 10,000 immigrant soldiers. This was over four days in January 1918. He was immediately asked to return to discuss ‘European Nationalities’.

He returned on January 24^th^ just four days after the War Department had offered honourable discharge to all those who requested it so that they did not have to fight against ‘fellow countrymen’ (German immigrants had been classed as ‘enemy aliens’ in December 1917). Despite being “then as anti-militarist as I am now” (Miller, 1926, Chap II, p. 7), Miller describes organising talks with men of different nationalities – Poles, Czechoslovaks, Roumanians and Jugoslavs. In an article, *‘America’s Lost Division’,* published in *The Survey*, he wrote that, “when it was explained to those men that America’s fight for freedom was the same as their own long struggle for freedom, the result was nothing short of amazing” (Miller, 1918a, p. 307). Nearly all refused the offer of discharge. Yet, the War Department compounded what Miller had thought to be their original error by declaring that they could serve only in non-combat roles, thereby indicating a continued prejudice against them, and providing them with tasks that were stigmatised by their association with fatigue duties or punishment.

Miller’s approach stressed *freedom from oppression* and its association with underlying and unrealised (American) values of freedom, not patriotism as such. Significantly, Du Bois expressed a similar ambivalence to the war, regarding participation in it as necessary to the furtherance of African American claims for equal citizenship. They should participate as Americans and, as participants, should be granted the same rights and respect as other citizens. As Karen Fields puts it, for Du Bois, “along with a double-consciousness went what can be called ‘double death’ – dying once for America and once for Afro-America” (Fields, 2012, p. 251). His experience of the failure of this strategy took him in more radical directions^35^, as it did for Miller with regard to his subsequent critique of the pathologies of nationalism. Miller advocated internationalism reconciled with a ‘proportional patriotism’: “It is my claim that already more than half of the values that give reality to our lives are internationally in existence and that the possibilities of pluralistic sovereignty make it entirely possible to be loyal to them. Most specific patriotic claims are anachronous” (Miller, 1921a, p. 143).

## ‘Forget my Future’

We know from Miller’s account that he became director of the Mid-European Union after a preliminary meeting of interested parties over a luncheon on October 3^rd^ 1918 where the participants were provided with two articles by Miller from *The Survey*, ‘The Bulwark of Freedom’ and ‘The Emergent Democracies’, as well as a map of European subject nationalities located in the territories of the ‘Central Powers’^36^. They resolved formally to establish the Mid-European Union. The formation was not an entirely happy one. Whereas President Wilson had been content to receive deputations from Masaryk (representing Czechoslovakia) and Paderewski (representing Poland), which Miller facilitated, there were complaints from the Italian ambassador that Miller was involving nationalities with claims upon each other’s territories (for example, Jugoslavia and territory claimed by Italy). He was told that he should cease his activities with the Union. However, Miller was also told by a secretary within the State Department that he should ignore that advice, that, “I ought to forget my future and do what I could” (Miller, 1926, Chap IV, p. 7).

Miller resolved to press on with gathering more representatives of oppressed peoples and nationalities within his idea for an organization that could contribute to a post-Imperial peace despite being told by the State Department to desist. Indeed, there is evidence (Fink, 2004; Gelfand, 1963) that, notwithstanding Woodrow Wilson’s pre-war commitments to self-determination, US policy toward a peace prior to entry in the war favoured an Austrian federation that recognized the rights of minorities rather than independence for small nations. Miller writes, “from that time on I was very much under official disapproval” (Miller, 1926, Chap IV, p. 10).

The Union prepared a declaration on behalf of oppressed peoples in Europe involving 12 signatories and space for more to be added. This was announced in *The Survey* (1918, p. ix) in glowing terms as something that, “might live in the text books of history after the week's ‘exits and alarums’ of military happenings had been relegated into the undated generalized background of the war^37^.” The statement was drafted by Paul Kellogg, who, in April 1918, had convened a small group to advocate for the rights of minorities, which would be designated six months later as the League of Free Nations (Chambers, 1971, pp. 72–3). Allen T. Burns was a member of the Founding Group and subsequently of the smaller executive committee^38^. Kellogg was disappointed by the direction of policy after the war and the compromise on the rights of minorities that came to be enshrined in the League of Nations when it was established in 1920 and the global claims of European empires were endorsed.

## Americanization and Differences Over its Methods

The founding of the Mid-European Union began a period of intense organisation which occasioned Thomas taking over Miller’s Division of the Carnegie project. The implication is that he had been too busy outside the project – and, indeed, had mis-directed his activities for some time, if Raushenbush is to be believed – but closer examination reveals that those activities were understood by Miller (and Burns) to be *part of the project*, not separate from it. Miller had also been involved in a hectic period of organisation under the initial authority of the US Government Committee of Information – a body to which Burns also reported – which involved contacting churches serving immigrant populations and benevolent societies, as part of the job of ‘filling the gap’ between Czechs and Jugoslavs by contacting Roumanian and Greek organisations (Miller, 1926, Chap III, p. 3)^39^.

*The Survey* – most likely, the editor, Paul Kellogg – introduced Miller’s article on ‘The Bulwark of Freedom’ with a description of its connection to the Carnegie project, writing that, “Professor Miller is one of the group brought together by Allen T. Burns in his survey of the problem of assimilation for the Carnegie Corporation. Through the cooperation of Professor Miller and Mr. Burns, readers of the Survey are getting the first fruits of their work in a series of articles…” (*The Survey*, 1918, p. x). Miller’s draft account of the period describes that in February he began his work on the Carnegie project, where his first task was to establish connections with the National Councils and influential individuals: “The Carnegie Corporation gave me a free hand to proceed as I wished” (Miller, 1926, Chap II, p. 8). Each week-end, after teaching, he comments, “I went to New York, Washington, or some army camp looking further into the situation among the soldiers” (Miller, 1926, Chap II, p. 8). In a letter in April to his brother-in-law, Paul Cravath, Miller wrote that, “you already know that I am helping in a national survey of Americanization under the Carnegie Corporation, and had been focusing on the Army side of it^40^.” For Miller, this involved making connections with all the immigrant groups associated with subject peoples – ‘filling the gap’ – and contributing to their formation into national organisations, with subsequent incorporation into the Middle European Union which was yet to be established.

Around this time, Burns was in a rather testy stand-off with the National Americanization Committee over its activities, together with the Bureau of Education, to establish a ‘Flag Day’ and ‘Loyalty Week’. The Carnegie Corporation had informed the Committee that it would no longer fund its work. The Committee had laid claim to the Carnegie Americanisation project as within its remit for promoting the war effort (as in Flag day), which Burns resisted firmly in correspondence with Henry Pritchett of the Carnegie Corporation, declaring the collaboration was instead aligned with the Committee on Information before concluding with the riposte, “you are probably acquainted with the small opinion of leading educators regarding work done by the Board of Education … This general opinion ought to be given due weight in deciding about an appropriation to an activity of the Board of Education^41^.” By the time the war had ended the balance had shifted back in favour of the National Americanization Committee and it was the Carnegie project on Americanization that was being side-lined.

Winifred Raushenbush’s later recollection of the peculiarities of the methodology adopted by Miller, then, is not so much incorrect as very partial. The methodology was possibly not that best suited to address immigrant heritages in normal times, but the times were not normal. Moreover, Miller’s emphasis on the ‘spirit of freedom’ that motivated immigrant communities and its alignment with American interests at war represented a more profound and radical contribution to understanding ‘Americanisation’ than that of the other contributions in the Carnegie project. It is, however, a contribution that has been lost to the history of sociology. When Thomas came to write *Old World Traits,* he provided no discussion of the research undertaken by Miller and his different understanding of Americanization organised in terms of immigrant heritages that expressed the aspirations of ‘oppressed’ peoples^42^. The Directors of the Divisions had permission to publish articles separately from the project reports and, in the case of Miller, this activity was of fundamental importance to the war effort and the aims of the Department of Information to which Burns reported. Miller published four articles derived from the Carnegie project during the period of the research (Miller, 1918a, b, c, 1919). Yet, when published, *Old World Traits* was but an addendum to the *Polish Peasant*, reinforced by the role of Florian Znaniecki as field researcher in replacement of Eleonor Ledbetter.

Thomas might have anticipated that his authorship would be recognised and would contribute to his rehabilitation, something that was blocked. However, Miller’s authorship credit reflected his initial role in the work – fully half of the period of the field research – albeit that the substance of his contribution was left out. The puzzle is Park’s authorship credit. Raushenbush does her best to justify it, but there is no evidence that he had any direct involvement at all^43^.

As we have seen, the American government wanted methods of Americanization that would be aligned with the country’s post-war foreign policy interests. This was something from which Miller had already indicated his divergence. Thomas delivered a manuscript that avoided the issue.

## Proportional Patriotism

Miller provided different conclusions to the study on Americanization in a series of articles written in the early 1920s, which drew on his research for the Carnegie project, but were not part of its report (Miller, 1919, 1921a, b, 1922). These articles were re-drafted and gathered into his book, *Races, Nations and Classes: The Psychology of Domination and Freedom* (Miller, 1924). The book was a sociologically informed discussion of its topics for a general audience to persuade them of the need for a rational program of freedom on a global scale.

Miller pointed out that “the intensity and diversity of opinion over the campaign for ‘Americanization’ have done much to stimulate thought. The Great War, the disorganization of Europe, the Ku Klux Klan and political restlessness have called attention to the need of a rational program rather than for a rationalized justification of status” (Miller, 1924, p. vii). If democracy is the answer, which Miller believed to be the case, then it is a pluralist democracy that recognised the self-determination of groups. This included, for Miller, the economic sphere and the functional representation of classes through industrial democracy. It will not have escaped notice that many of the groups identified by Miller are those that were the focus of the Carnegie project. It is clear from Miller’s book that he intended it as a riposte, since his penultimate chapter is entitled the ‘paradox of Americanization’ with the conclusion called ‘proportionate loyalty’.

This is the basis of ‘proportional patriotism’; the ‘internationalisation’ of groups, including classes, who become the basis of the human values that embed local democracies. Initially, Miller sets this out in a manner reminiscent of the Government’s Memorandum on Americanization, only to represent what the latter perceived to be the risk as, in fact, the solution. He writes, “the foreign-born will never forget the land of their origin and their responsibility for it so long as injustice prevails there; the identification of America with the problems of Europe, therefore, is so close that we cannot escape our share in the responsibility however we may wish. There can be no real Americanization of the immigrant unless there is a real league of nations, as the symbol of a real organization which will substitute in Europe a reign of justice for the reign of immorality” (Miller, 1924, p. 177).

In his *Races, Nations and Classes*, Miller developed a global perspective on domination and oppression. With regard to Americanization, both in the Cleveland survey and in the Carnegie project, Miller advocated cultural pluralism and the independent contribution that immigrant cultures might make to the self-understanding of the idea of America, otherwise expressed in a problematic and unselfconscious ‘Anglo-Americanism’. This was a significant part of his approach to the direction of his Division of the Carnegie study^44^. His methods of Americanization differed significantly from the adopted ‘two-step’ approach toward assimilation in which immigrant organizations served to integrate immigrants into a collective adaptation to American life before a more complete assimilation (Smith, 1939). ‘Forget your future’, Miller was told. It was not just his political future that was stymied, also forgotten was his valuable contribution to the study of immigrants, oppressed groups, races and nations. They remained on the fringes of sociological interest.

## Conclusion

In this article, we have presented Miller’s approach to issues of race and immigration through the lens of subject minorities and nationalities in the context of the Austro-Hungarian empire and its alliance with the German empire in the first world war. We have not had the space to consider how Miller also understood African Americans to be subject to oppression. This will be the topic of a second paper, which will also discuss Miller’s role as one of the first critics of eugenics within US social science. Indeed, his engagement – practical as well as theoretical – with race relations in the US was a constant feature. In 1924–5, he was drawn into a dispute at Fisk University when he was appointed as chair of a committee to resolve the future of the university after student protests had forced the resignation of its President, Lafayette McKenzie (Lamon, 1974; Taylor, 1952). Indeed, it was after his intervention–that the ground was set for the appointment of Charles S. Johnson, who came to be regarded as the most important sociologist at Fisk and would become its first Black president in 1946.

Nor was Miller’s concern with the domination and oppression associated with European empires confined to minorities within Europe. He was a critic of British empire and an early advocate of non-violent resistance in India. By the same token, he was also a critic of Japanese imperialism, especially in Korea. In a very ambitious way, Miller identified a new enriched global order that would be brought into being by the revolution of ‘colored peoples’ against empires and their embedded justifications of domination through ideas of racial superiority. It is this combination of interests and practical engagements, sociological as well as political that would lead to his dismissal from Ohio State University in 1932, coinciding with the delivery to the press of his last book, *The Beginnings of To-Morrow* (Miller, 1933) in which these arguments were articulated. The reasons given were various – his advocacy in practice, as well as in theory, of race-mixing, and his criticisms of British empire.

Miller is a largely forgotten figure, but he was a pioneer of a global sociological perspective on domestic issues of race and ethnic relations. His sociological approach expressed the pragmatist concern with science *and* citizenship. He made a distinctive contribution to the sociological science of race and ethnic relations, while his concern for the rights of all citizens operated on the most expansive canvas of global justice.

## Footnotes

1 This article was written as part of the research project: “Rethinking Domination” (grant no. GA20-28212S), funded by the Czech Science Foundation. It was written during the Covid-19 epidemic when libraries and archives were closed to visitors. We are grateful to archivists et al. who were willing to retrieve and copy material for us. In particular, we thank, Leah Richardson, Special Collections Librarian, George Washington University; Louisa Hoffman, Oberlin College Archive; Plummer ‘Al’ Jones Jr, East Carolina University; Phillip Cunningham, Head of Research Services, Amistad Research Center, Tulane University; Robert Spinelli and Brynna Farris, John Hope and Aurelia E. Franklin Library, Fisk University, John Hope and Aurelia E. Franklin Library, Special Collections; Margery E. Sly Temple University; Vanessa Lee, Rare Book & Manuscript Library, Columbia University in the City of New York.

2 Interestingly, Mills (1943) wrote an article criticising ‘social pathologists’ in which Miller is mentioned in three footnotes (albeit in one, it is to the editor of the series in which Miller’s book on *Races, Nations and Classes* (Miller, 1924) was published). ‘Social pathologists, according to Mills, operate from a perspective of ‘social disorganisation’, a term repudiated by Miller as we shall see. Mills also argues that the social pathologists fail to address large social structures, when Miller patently is concerned with the structures of domination associated with race, class and nation. The viewpoint that Miller was working on large structures is confirmed by Luther L Bernard and Jessie Bernard in their survey of sociological contributions to international relations. For Miller, as they put it, nationalism, “is essentially a revolt against political and cultural imperialism. It flourishes to best advantage under repression, and as a consequence it develops an oppression psychosis. He believes it to be a disease, but thinks it must run its course and, … that its cure will be the disappearance of imperialism” (Bernard & Bernard, 1934, pp. 63–4). Mills does not include W.I. Thomas in his strictures about social pathologists, notwithstanding that the latter does operate with a concept of social disorganisation brought about by the migrant process.

3 Before his appointment at Oberlin, Miller taught at its ‘sister’ college in rural Michigan, Olivet between 1905 and 1914. As part of his pedagogic practice throughout his career, Miller organised classes together with students at the ‘historically Black’ college, Wilberforce, as well as continuing with his involvement at Fisk.

4 Descriptive Inventory of the Personal Papers of Herbert Adolphus Miller: Biographical Note. Temple University, Samuel Paley Library, Conwellana-Templana Collection. It is also confirmed by Miller in his ‘memoirs’ albeit without dates. In a letter from Moscow to his wife in 1925, Miller also recalls an earlier trip to Moscow in 1912 as part of the same trip. Letter from Miller to Miller, Temple University, Samuel Paley Library, Conwellana-Templana Collection, Folder D. Mary E. McDowell provided introductions to Thomas Masaryk and Alice Masaryk in Prague.

5 Letter from Thomas to Young, May 4, 1930. Cited in Chapoulie (2020, p. 374, n. 178). Thomas had been forced to resign from the University of Chicago in April 1918, following a scandal involving the wife of an officer serving overseas. Charges against Thomas under the Mann Act were subsequently dropped.

6 The suspicion that Miller had been disliked at Oberlin and “was about to lose his position there” (Sabine, 1931, p. 469) featured prominently on the list of reasons given by the Ohio State University’s Board of Trustees to justify the dismissal of Miller. The president of Ohio State himself visited Oberlin and personally conceded to Miller that “no question had ever been raised in any way as to my dismissal from Oberlin.” Letter from Miller to Tyler, May 26, 1931. Cited in Sabine (1931, p 467).

7 The *Bulletin of the Ohio Sociological Society*, did not publish in 1931 and its first issue of 1932 – volume V(I) – published in May out of the Ohio State University Sociology Department ran to just 3 pages of news. It reported laconically that, “Dr J. E. Hegerty has resigned as head of the Department of Sociology at Ohio State after thirty years of active service.” Hegerty was very active in defence of Miller, but it seems that the latter was stoic in not using his presidential position to advocate in his own cause. The *Bulletin* is archived on Jstor.

8 Kellogg’s very considerable flair probably contributed to the idea of the *Survey* magazine being more about ‘publicity’ (in the best and pragmatist sense of that word) rather than ‘science’, notwithstanding the research basis of its articles (Chambers, 1971).

9 This was a mass killing of miners and their families by the state national guard and company militia on April 20^th^ 1914 during protracted strikes on the Colorado coalfields that took place across 1913–1914.

10 “To Investigate all Foundations,” *New York Times*, December 17, 1914, p. 6.

11 It was exacerbated by the fact that evening classes for adults were funded on the basis of enrolment, rather than attendance – by the end of a term only about one fifth of the enrolled class would be in attendance (Miller, 1916, pp. 98–100).

12 Grace Abbott, for example, who would also be appointed to the project to undertake a study of the immigrant and the courts (she later had to withdraw because of ill health) had earlier affirmed Miller’s arguments in her study, *The Immigrant and the Community*: “In our zeal to teach patriotism, we are often teaching disrespect for the history and tradition that the ancestors of the immigrant parent had their part in making … this often means disrespect for the parent himself”(Abbott, 1917, p. 226).

13 Americanization Studies, 1918–1945. Carnegie Corporation of New York Records. Rare Book and Manuscript Library. Columbia University Libraries. [Series III: Grants, 1911–1994., Subseries III.A., Box 41, Folder 4–6].

14 A shortened version was published under a different title in the *Annals of the American Academy of Political and Social Science* (Burns, 1921).

15 The review committee – Talcott Williams, Raymond B. Fosdick, Dr Edwin F Gay – evidently regarded some of the studies too partisan. Leiserson was a progressive economist who would go on to be an adviser to Franklin D. Roosevelt.

16 The Carnegie Corporation Archive on the Americanization project indicates that reviewers thought that some of the books in the series were of ‘uncertain quality’ which delayed the publication of the set. Burns indicated that he had a summary report available to publish in January 1924, but by then the Corporation was in discussion with the publishers about ceasing publication.

17 Burns left the employment of the Corporation. His career then turned almost exclusively toward work for charitable bodies. After a short interlude as Chair of the Labour Adjustment Board of the Rochester, N.Y, clothing industry, he was appointed in 1922 as executive director of the National Information Bureau, advising on charitable appeals, as part of which he went to Russia (together with Graham Taylor) as part of a three-person Commission on Russian Relief. He held this position in combination with the American Association for Community Organizations which was linked with the Bureau. In 1926 he became executive secretary of the successor and independent body, the Community Chests and Councils of America Inc, in 1926 and continued with that organization until his retirement in 1942. See Friends and Relatives of Allen T. Burns (1954).

18 It is discussed more fully in Smith (1939) *Americans in the Making: The Natural History of the Assimilation of Immigrants*. As might be anticipated from the sub-heading, Smith adopts the Chicago school approach in outlining a two-stage process of assimilation and aligns *Old World Traits* with that argument. .

19 There is no discussion of the episode in the material collected in the Carnegie Corporation archive on the Americanization project. The exchange of letters takes place in November 1951 when Burgess refers to a ‘recent’ meeting in St Louis with Burns when he discussed the matter and asks him for an ‘authoritative statement’ in one sentence for the publication committee, that the book was “actually written by W.I. Thomas, but for reasons that do not need to be entered into, it appeared under the name of Park and Miller” (Burgess to Burns, Nov 22 1949). Burns replies, “How’ll this do. The volume Old World Traits of the Americanization Studies was primarily written by W.I. Thomas though at the time it was considered to have it appear under the authorship of Park and Miller who also worked on the volume. I am very glad that Prof Thomas is to receive credit for his invaluable contribution” (Burns to Burgess, Nov 24, 1949). Burgess, Ernest. Papers, [Box 3, Folder 3], Hanna Holborn Gray Special Collections Research Center, University of Chicago Library.

20 It seems most likely that it was at the behest of the publisher whose concerns about sales of all the books in the series is recorded throughout the Carnegie Corporation files on the Americanization project. Certainly, the report was delivered in December 1919 under the name of Thomas, recorded in the minutes of the Conference of the advisory committee, which included Henry S. Pritchett, the President of the Carnegie Corporation. A contract with Harper and Brothers was signed by the Vice Chairman and Treasurer of the Corporation was signed on June 7^th^ 1920 with the names of Robert E. Park and Herbert A. Miller entered as authors. Americanization Studies, 1918–1945. Carnegie Corporation of New York Records. Rare Book and Manuscript Library. Columbia University Libraries. [Series III: Grants, 1911–1994., Subseries III.A., Box 41, Folder 4–6].

21 See, for example, Chapoulie (2020, pp. 374–375, note 148). In fact, though she does not mention it, she had been taught by Miller at Oberlin and, on her graduation in 1916, he had recommended her for a teaching position at a historically Black college (most probably, Wilberforce, with which he also had a connection), which her father had not wanted her to take up. See Gross (2008, p. 68, n. 18).

22 Abbott and Egloff (2008, p. 221) comment that, “Thomas's work for the Carnegie Foundation antedated the episode and thus was not cooked up by his friends to give him work, as has been sometimes assumed”. Letter from Thomas to Dummer, April 9, 1918. In Thomas’s testy exchange with Kimball Young over his biography some truth to the standard version is conceded: “You know darned well how much I had to do with ‘Old World Traits Transplanted,’ but do you expect me to tell you for publication? The men concerned there were my friends, and acted friendly. I needed the money” (cited in Throop & Ward, 2007, n.p.).

23 Americanization Studies, 1918–1945. Carnegie Corporation of New York Records. Rare Book and Manuscript Library. Columbia University Libraries. [Series III: Grants, 1911–1994., Subseries III.A., Box 41, Folder 4–6].

24 An extra subvention of $5000 is recorded in the October minutes of the 6^th^ Conference of the advisory committee, but this attracts the note, ‘on the grounds that the importance and extent of inquiry require $5,000 additional appropriation’. Americanization Studies, 1918–1945. Carnegie Corporation of New York Records. Rare Book and Manuscript Library. Columbia University Libraries. [Series III: Grants, 1911–1994., Subseries III.A., Box 41, Folder 4–6].

25 Al Jones kindly provided us with a copy of her letter to the Librarian at Cleveland Public Library, where she wrote, “I submitted to [Mrs Horvath] Mr Park’s outlines of the work he wants done. Mrs Horvath, as an immigrant felt about them exactly as I had expected, and said she would be much offended if I, as an investigator, were really to ask her those questions. I was gratified to have this confirmation of my own judgement, which satisfied me completely that I have done well in resigning. Now to get back home.”.

26 Americanization Studies, 1918–1945. Carnegie Corporation of New York Records. Rare Book and Manuscript Library. Columbia University Libraries. [Series III: Grants, 1911–1994., Subseries III.A., Box 41, Folder 4–6].

27 A shorter version of a similar survey is presented at the end of an earlier article by Thomas (1912) on ‘race psychology’ which serves as a model for the document presented to inform the study of immigrant heritages.

28 Letter in January for meeting Feb 15^th^. Americanization Studies, 1918–1945. Carnegie Corporation of New York Records. Rare Book and Manuscript Library. Columbia University Libraries. [Series III: Grants, 1911–1994., Subseries III.A., Box 41, Folder 4–6].

29 In 1902, Thomas Masaryk visited the University of Chicago at the invitation of the president William R. Harper who offered him 2 000 USD for a series of 24 lectures for non academic audience. The number of attenders fluctuated between 40 and 60. Later, Masaryk summarized the main topic of his Chicago lectures as “the philosophy of the history of a small nation” (see Kovtun, 1988, p. viii).

30 Miller went to Bohemia in 1912 with a large delegation of American Bohemians. He spent three weeks in Prague and received an invitation to spend a week with Masaryk who was then spending his summer vacations in Moravia. Miller also names Mary McDowell as the person who helped him establish contacts with the Masaryks (Miller, 1940, p. 74).

31 In a letter dated July 12, 1926, Miller writes that he has just sent off the completed manuscript of his story “An American Sidelight on the Czechoslovak Revolution”, indicating that it had been due in Prague the first of June for translation and publication during the summer. Letter from Miller to Hinman, July 12, 1926. Herbert A. Miller Papers, Folder Correspondence—Miscellaneous, Box 1, Oberlin College Archives RG 30/23.

32 See Švec (2007, p. 316) and Letter from Miller to Masaryk, July 17, 1926, Correspondence, Folder 58. Thomas Garrigue Masaryk Archive.

33 It is referenced by Teeters (1951) in his obituary of Miller and he deposited a set of papers associated with Miller in an archive at Temple University (together with notes of areas where it seemed to have missing pages. Teeters (1966), wrote a short summary of the memoir for the *Hartwick Review*, but it contains no details of Miller’s account of the relation between Miller’s work with the Mid European Union and the Carnegie project. There is archival evidence that Miller sent his autobiography to Luther Bernard for his project on the history of sociology in the U.S. The file with Miller’s contribution, stored at the University of Chicago Library, contains only a letter from Bernard to Miller, appreciating his “very interesting life history”. Luther Lee Bernard Papers 1928–1938, Series II: Life Histories of Sociologists, Box 2, Folder 19. The text itself is missing. The main Luther Bernard Collection at Pennsylvania State University Library gives out only a short excerpt by Bernard from Miller’s work.

34 The statement is characteristically modest. He appears in all the letters to the Austrian Ambassador as joint signatory. Herbert A. Miller Papers, Series I Correspondence, Box I, Folder – Correspondence between Miller and Woolsey, 1916, Oberlin College Archives RG 30/23. The larger network of campaigners comprised very prominent friends of the Masaryks such as Charles Crane, Julia Lathorp, or Adolph Sabath (see Crawford Mitchell, 1980, pp. 85–91).

35 In his later autobiographical reflections on this time, Du Bois wrote, “I am less sure now than then of the soundness of this war attitude. I did not realise the full horror of war and its wide impotence as a method of social reform. Perhaps, despite words, I was thinking narrowly of the interests of my group and was willing to let the world go to hell if the black man went free … Possibly passive resistance of my twelve millions to any war activity might have saved the world for black and white” (Du Bois, 1995[1940], pp. 407–408).

36 The Central Powers were those of the German Empire, the Habsburg dual monarchy of Austro-Hungary, the Ottoman Empire and the Kingdom of Bulgaria. It is this map that is reproduced in the first edition of *Old World Traits.*

37 They also referred to an earlier body set up in 1917, the League for Small and Subject Nationalities. This was chaired by Cleveland Senator, Frederic C. Howe, and had been subject to press vilification for being pro-German, which was part of the context to Miller’s trip to Camp Sherman.

38 Its founding is described in the Survey by Kellogg, together with the ringing statement, “we stand for absolute freedom of religion, of civil liberty, of cultural development of the weaker peoples within the stronger nations, and of the native peoples of the undeveloped regions of the earth” (Kellogg, 1918, p. 251).

39 In its context, Miller meant making connections between the middle-European countries and those further East and South.

40 Letter from Miller to Cravath, Masaryk papers, National Archive, London. April 29, 1918. Indeed, the March minutes of the Carnegie advisory committee describing the appointment of the heads of the ten divisions notes that Miller was a professor at Oberlin College and ‘Military Adviser, Camp Sherman’. Americanization Studies, 1918–1945. Carnegie Corporation of New York Records. Rare Book and Manuscript Library. Columbia University Libraries. [Series III: Grants, 1911–1994., Subseries III.A., Box 41, Folder 4–6].

41 Letter from Burns to Pritchett, May 6, 1918. The formal collaboration appears to have been with the Division of Work With the Foreign Born of the Committee on Public Information and continued until May 1919. Americanization Studies, 1918–1945. Carnegie Corporation of New York Records. Rare Book and Manuscript Library. Columbia University Libraries. [Series III: Grants, 1911–1994., Subseries III.A., Box 41, Folder 4–6].

42 The publication of the book was accompanied by a frontispiece map. ‘The Peace Treaty is an attempt to make racial and political boundaries more nearly coincide’, as an echo of Miller’s interests.

43 The republication of the American Studies series of books in 1971 occurs with the author credit as, ‘William I. Thomas, together with Robert E. Park and Herbert A. Miller’.

44 There is indirect evidence that it was the view of Burns (1921), too, as set out in his paper, ‘Immigrants, the matrix of democracy’.
